# Real-world evidence from a multi-center pediatric network: greenness exposure and autism spectrum disorder in urban China

**DOI:** 10.3389/fpubh.2025.1666873

**Published:** 2025-09-17

**Authors:** Wenxu Yang, Shiwen He, Sophia Zuoqiu

**Affiliations:** ^1^Department of Child Healthcare, Chengdu Women and Children’s Hospital, School of Medicine, University of Electronic Science and Technology of China, Chengdu, China; ^2^Department of Environmental Science and Engineering, College of Architecture and Environment, Sichuan University, Chengdu, China; ^3^Department of Industrial Engineering, Pittsburg Institute, Sichuan University, Chengdu, China

**Keywords:** real-world data, child development, greenness exposure, childhood ASD prevalence, NDVI

## Abstract

**Introduction:**

Childhood autism spectrum disorder (ASD) is a global public health concern and its prevalence is increasing rapidly in developing countries such as China. The mechanism behind ASD development remains unclear. Greenness exposure is reportedly associated with various health outcomes, however, the connection between greenness exposure and ASD is relatively unexplored.

**Methods:**

We designed a two-stage screening process and conducted city-wide screening for early childhood ASD to investigate the association between greenness exposure and ASD prevalence in a megacity in southwest China.

**Results:**

We screened 13,458 children from 0–52 months through 20 local primary care hospitals and the estimated ASD prevalence was 0.55%. We matched greenness exposure, air pollution exposure, and weather condition with ASD diagnosis outcomes based on the study subject’s geographic information. Gender (male) and age (older) were significantly associated with higher odds of being diagnosed with ASD.

**Discussion:**

Although the association between ASD diagnosis and greenness was not statistically significant, real-world data may help improve ASD screening methods and guide future studies. Our findings highlight the potential role of real-world environmental and health data in informing sustainable urban and pediatric health policies.

## Introduction

Autism spectrum disorder (ASD), a neurodevelopmental disorder disease, is characterized by chronic social difficulties and repetitive stereotyped interests or behaviors emerged in early childhood (1, 46, 52). Children with ASD exhibit poor ability for social interaction and have difficulties integrating into society (2). Moreover, autism patients usually experience increased financial burden as they and their families need to pay for diagnosis, treatment, and special education (54). In the past 30 years, ASD prevalence has risen sharply around the world (50). It is estimated that about one in every 100 children worldwide has autism. However, this estimate is an average figure, and reports of autism prevalence vary widely across studies (3). The estimated ASD prevalence rates appeared to be higher in more developed and urbanized regions in the world, such as Europe, the Middle East, Australia and New Zealand, and Central and South America, while Africa was not clearly documented (4, 5). ASD prevalence in China has demonstrated similar upward trend due to a combination of increasing awareness, improved diagnostic tools, and extensive research efforts (6). Studies have shown that pooled ASD prevalence estimates have increased over time. The pooled prevalence estimate increased from 8.5 per 10,000 population (range: 3.0, 13.9) between 2000 and 2004 to 16.4 per 10,000 population (range: 7.0, 25.7) between 2010 and 2011 (7). A large-scale study conducted in 2019 in mainland China found that the prevalence of ASD was approximately 1 in 100 children, or about 1% (8). The reported prevalence rates were similar in many Western countries, but it reflects a significant increase compared to earlier estimates. A recent investigation indicated the ASD number was 26.2/1000 among 3–4 year-old children in Shenzhen in 2020 (4).

To date, scientific understanding of the mechanism behind ASD development remains limited (45). A number of studies explored the relationship between exposure to nature (or greenness exposure) and ASD (1, 9, 10), but the findings were inconclusive on the beneficial effect of greenness exposure. Some early studies on greenness exposure suggest a potentially positive impact on various health outcomes (11), including depressive disorders and ASD (9, 12). While findings from these studies provided early evidence about childhood ASD in connection with greenness exposure, these studies were mostly conducted in developed countries of the world with a focus on older children (age 5 and above). Currently, very limited information is available on the effect of greenness exposure on ASD prevalence in developing regions and among younger children (age 5 and below).

To better understand the potential relationship between greenness exposure and childhood ASD, we collected real-world data and conducted an explorative investigation on the potential relationship between urban green space and early childhood ASD detection. We utilized local health service network in Chengdu, the capital city of Sichuan Province of southwestern China, to conduct early screening for children aged 0–52 months from 20 districts or district-level counties for an explorative investigation on the relationship between greenness exposure and ASD prevalence. Based on existing literature, we hypothesize that higher greenness exposure is associated with lower probability of ASD diagnosis. The findings based on real-world evidence from this study could provide scientific basis for resource allocation for early ASD screening and could help to mitigate the economic burden caused by ASD at both individual and societal levels.

## Research methods and materials

### Recruitment, screening and diagnosis

Children between 0 and 52 months of age routinely visit the local maternal and child care hospitals for child health care check, and we screened children during such visits between Oct 1, 2020 and May 31, 2021 in Chengdu, China, through local maternal and child healthcare hospital network. A total of 20 maternal and childcare primary hospitals located in 20 administrative districts/counties participated in this study; for a full list of the participating hospitals, please see Supplementary Table S1 in Supplemental Information. Population distribution by district/county was obtained from publicly available Seventh National Census data (13, 14). All parents (or guardians) provided consent to participate in the study. This study was approved by the Ethics Committee of the Chengdu Women’s and Children’s Central Hospital, School of Medicine, University of Electronic Science and Technology of China (2020–98).

**Figure 1:**
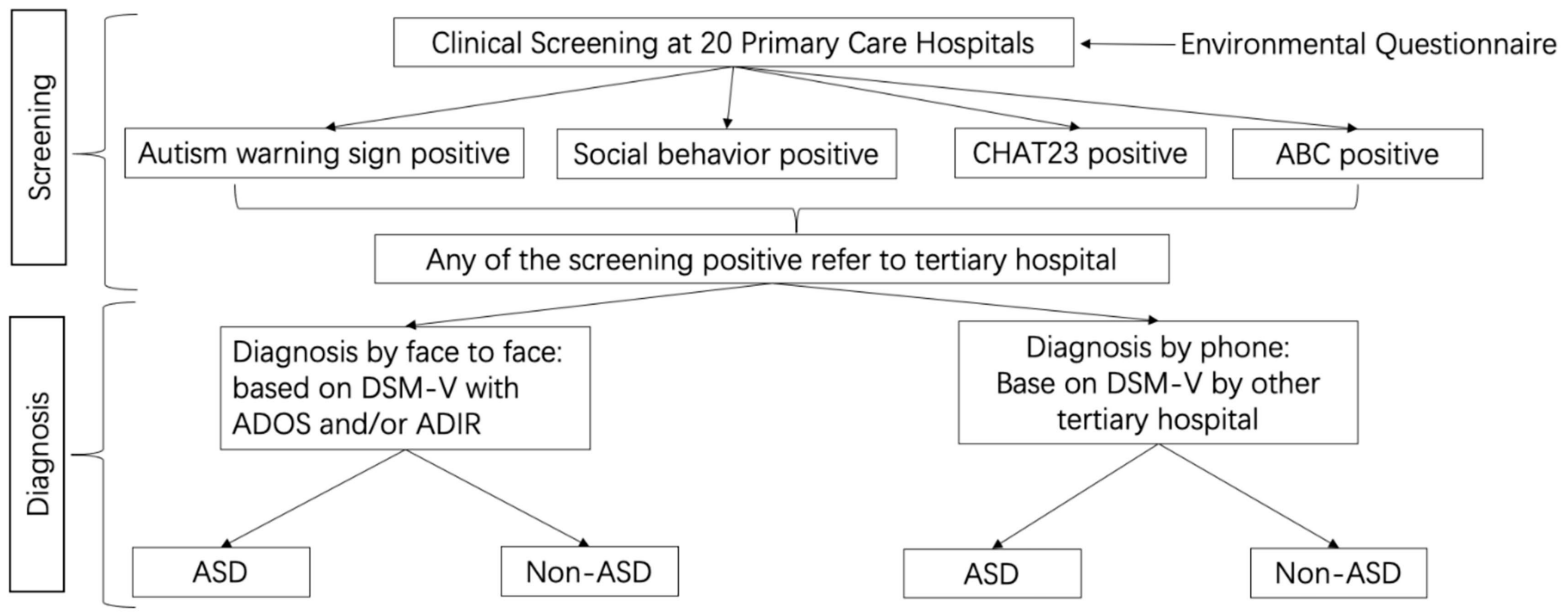
Two-stage screening process with initial screening at primary care hospitals and confirmation diagnostic assessment at tertiary hospital.

A two-stage screening process consisted of initial screening at primary care hospitals and confirmation diagnostic assessment at tertiary hospital (Figure 1). Initial screening for children 0–52 months utilized four screening methods autism warning signs, social behavior observational assessment, Chinese-validated version of the Checklist for Autism in Toddlers (CHAT-23), and autism behavior checklist (ABC). The target age range for analysis is 18 to 52 months (inclusive) because the screening tools are most suitable for children 18 months and older. For information on the selection of these screening tools and their respective clinical references, please see Supplemental section (Supplementary Table S2). A positive result from any one of the four screening methods was defined as a positive case. A secondary assessment by a different doctor was performed on identified cases. An affirmative (positive) assessment result by the secondary assessor leads to a referral to tertiary hospital, whereas a negative assessment result by the secondary assessor requiring further assessment were recorded as non-case for the purpose of this study. Confirmation diagnoses at tertiary hospitals utilized gold standard diagnostics tools [Autism Diagnostic Observation Schedule (ADOS) or/and Autism Diagnostic Interview-Revised (ADI-R)] based on the Diagnostic and Statistical Manual of Mental Disorders, Fifth Edition (DSM-5). The tertiary hospital assessors were blind to the screening status. We carried out monthly quality control assessment for both the clinicians and on data collected. Specifically, we (1) checked in with clinicians to review the screening procedure and to discuss problems in conducting the screening, and we (2) reviewed data for issues such as inconsistency and missingness. All identified issues were resolved promptly.

### Greenness exposure

We distributed environmental questionnaires during the ASD screening process through the 20 participating primary hospitals. Participation in the environmental questionnaire was voluntary. The environmental questionnaire included information such as the child’s name, gender, date of birth, primary hospital, and home address. Home address was used to extract the latitude and longitude coordinates for matching with residential outdoor vegetation coverage, which reflects the level of greenness exposure.

Greenness exposure is commonly quantified by Normalized Differential Vegetation Index (NDVI), which estimates vegetation coverage (Fractional Vegetation Coverage, FVC). FVC specifically expresses the percentage of the vertical projected area of vegetation (including leaves, stems and branches) on the ground to the total area of the statistical area (15, 16). NDVI quantifies vegetation by measuring the difference between near-infrared (which vegetation strongly reflects) and red light (which vegetation absorbs) (Equation 1). Thus, the value was calculated from the surface reflectance in the near-infrared and infrared bands according to a fixed formula (17, 18). The surface reflectance in the near-infrared and infrared bands comes from the Landsat 8 launched by NASA, Band 5, and Band 4, respectively. The formula for calculating NDVI is:


(1) $$\text{NDVI}=\frac{\mathrm{NIR}-\mathrm{Red}}{\mathrm{NIR}+\mathrm{Red}}$$

Where NIR and red represent the reflectance of near-infrared band and red band, respectively.

Vegetation coverage can be calculated by a pixel binary model. The pixel binary model divides the pixels into two categories: bare soil and pure vegetation. The actual NDVI value of the target pixel is the combination of pure vegetation and bare soil in a certain proportion (Equation 2), namely:


(2) $$\text{NDVI}=\mathrm{FVC}\times {\text{NDVI}}_{\mathrm{veg}}+(1-\mathrm{FVC})\times {\text{NDVI}}_{\text{soil}}$$

Where NDVI is normalized difference vegetation index for the target pixel, FVC is fractional vegetation coverage, NDVIveg is NDVI for full vegetation pixel based on the pixel binary model and NDVIsoi is NDVI for bare soil coverage pixel. For detailed information on FVC calculation, please refer to Supplementary material.

According to the calculation method of vegetation coverage, the data with cumulative probability of 1 and 99% were selected as NDVImin and NDVImax, and NDVImin = 0.195 and NDVImax = 0.864. The value of NDVI ranges from −1 to 1: when there is vegetation cover, NDVI is positive. When the ground cover is rock or bare ground, NDVI is close to 0. When the ground cover is obstructed by cloud, or covered by water or snow, the calculated value is negative.

We matched annual mean NDVI with coordinates of study subject’s residential address and created greenness exposure buffers at the following radii: 50 m, 100, 500, and 1,000 m, using the spatial mean NDVI within the buffer range as a supplementary parameter.

### Surface vegetation type

We obtained land cover types in Chengdu from 2018 to 2021 from annual land cover data of China released by Wuhan University (19) and then used ArcGIS to map surface vegetation for the study region (20).

### Air pollution and weather condition

Because previous studies have reported potential relationship between air pollution exposure and ASD development in children (21–23, 48), we included air pollution data in our anlaysis to adjust for potential confounding. Hourly concentration data of six major air pollutants (PM_2.5_, PM_10_, O_3_, NO_2_, SO_2_, and CO) were retrieved from the China National Environmental Monitoring Centre (CNEMC) China National Environmental Monitoring Centre (24). Hourly raw meteorological data (ground temperature and relatively humidity) with a spatial resolution of 0.25° were obtained from the National Oceanic and Atmospheric Administration, or NOAA (25). Grid with 0.01°X0.01° resolution was created and then aligned the environmental data; individuals’ addresses were subsequently matched with the predefined grid. Daily PM_2.5_ (μg/m^3^), PM_10_ (μg/m^3^), O_3_ (μg/m^3^), NO_2_ (μg/m^3^), SO_2_ (μg/m^3^), and CO (mg/m^3^) concentrations, as well as daily ground-level temperature (°C) and relative humidity (%), were estimated to match the address grids. We inputted air quality measurements, satellite retrievals, and various auxiliary variables such as meteorological conditions, population density, and land use types into six separate machine learning models (i.e., extreme gradient boosting) to estimate the gridded concentrations of each air pollutant. These machine learning models were utilized in previous studies to fill in measurement gaps. For details of the modeling process and predictive performance, please refer to work done by Zhan et al. (26–28). Person-specific air pollution and weather data were included in the analysis as potential confounders.

### Statistical analysis

SPSS 24.0 statistical software was used to summarize and analyze data (29). The main logistic regression model was constructed in R language (R-Forge Team., 2016) to analyze the potential relationship between greenness exposure (as represented by NDVI) and ASD diagnosis. ASD diagnosis outcome (positive or negative) was the outcome variable of interest (dependent variable) and person-specific annual mean NDVI was the primary exposure variable of interest (independent variable). The model was adjusted for the children’s demographic information (age and gender), person-specific air pollution based on address-matched annual mean concentration of PM_2.5_, PM_10_, O_3_, SO_2_, NO_2_, CO, and local weather parameters (e.g., annual mean temperature and relative humidity). Additional analysis was conducted by age stratification. An additional model was constructed with mean NDVI at circular buffers with radii of 50 m, 100 m, 500 m and 1,000 m.

## Results

### Population characteristics and ASD prevalence

**Figure 2:**
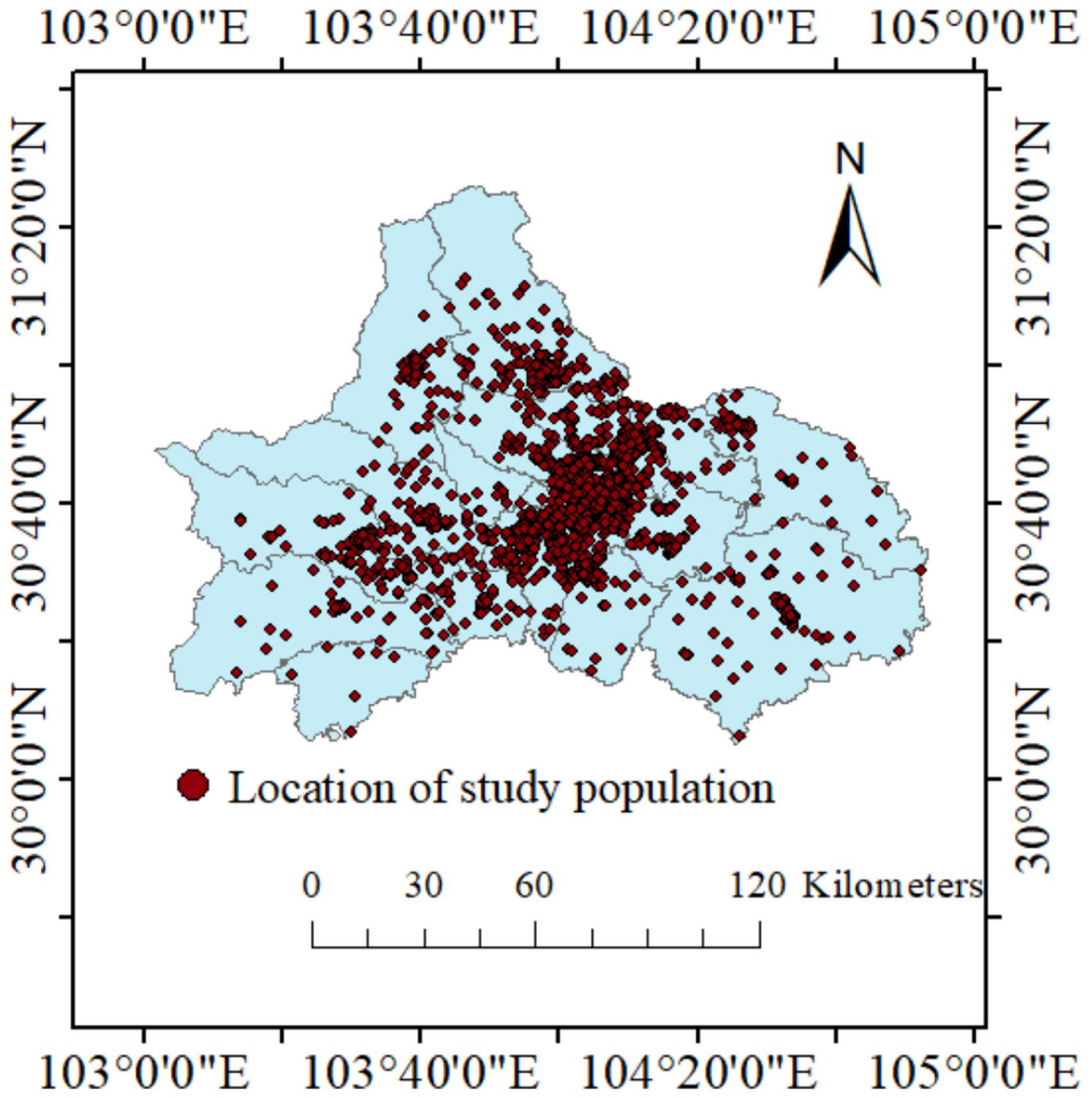
Location of study population in Chengdu, Sichuan Province. Red dot represents each study individual.

We screened a total of 13,458 children aged 0–52 months through 20 local maternal and childcare hospitals. Analysis on greenness exposure included 4,362 individuals who provided complete clinical data and environmental questionnaire results, and most of them were between the age of 13 and 24 months (88.58%) (Table 1). The study population included slightly more boys (51.47%) than girls (48.53%) (Table 1). In terms of distribution area, most of the children lived within urban core of Chengdu, and the rest lived throughout all districts of the city (Figure 2).

**Table 1:** Summary of study population and ASD cases.

District	*N*	ASD (%)
Chenghua	439	1 (0.23)
Chongzhou	113	0 (0.00)
Dayi	377	0 (0.00)
Dujiangyan	85	1 (1.18)
Jianyang	174	1 (0.57)
Jinniu	429	3 (0.70)
Jintang	136	1 (0.74)
Jinjiang	112	1 (0.89)
Longquanyi	106	1 (0.94)
Pengzhou	352	3 (0.85)
Pidu	136	0 (0.00)
Pujiang	4	0 (0.00)
Qingbaijiang	46	0 (0.00)
Qingyang	21	0 (0.00)
Qionglai	50	0 (0.00)
Shuangliu	757	4 (0.53)
Wenjiang	10	0 (0.00)
Wuhou	197	1 (0.51)
Xindu	761	7 (0.92)
Xinjin	57	0 (0.00)
Total	4,362	24 (0.55)

Gender	*N*	ASD (%)
Male	2,245	15 (0.67)
Female	2,117	9 (0.43)

Age (months)	*N*	ASD (%)
0–12	59	0 (0.00)
13–24	4,103	23 (0.56)
25–36	194	1 (0.52)
37–52	6	0 (0.00)

N is number of unique individuals.

Out of the 4,362 children included in the analysis, 24 were diagnosed with ASD and the estimated ASD prevalence is 0.55% (Table 1). The incidence of ASD also varies between regions. As the largest proportion of study population of Xindu District, it also had the highest number of ASD cases (7 cases). Additionally, districts Shuangliu (4 cases), Jinniu (3 cases), Pengzhou (3 cases), Dujiangyan (1 case), Jianyang (1 case), and Longquanyi (1 case) all reported ASD positive cases.

### Temporal and spatial variability in NDVI

**Figure 3:**
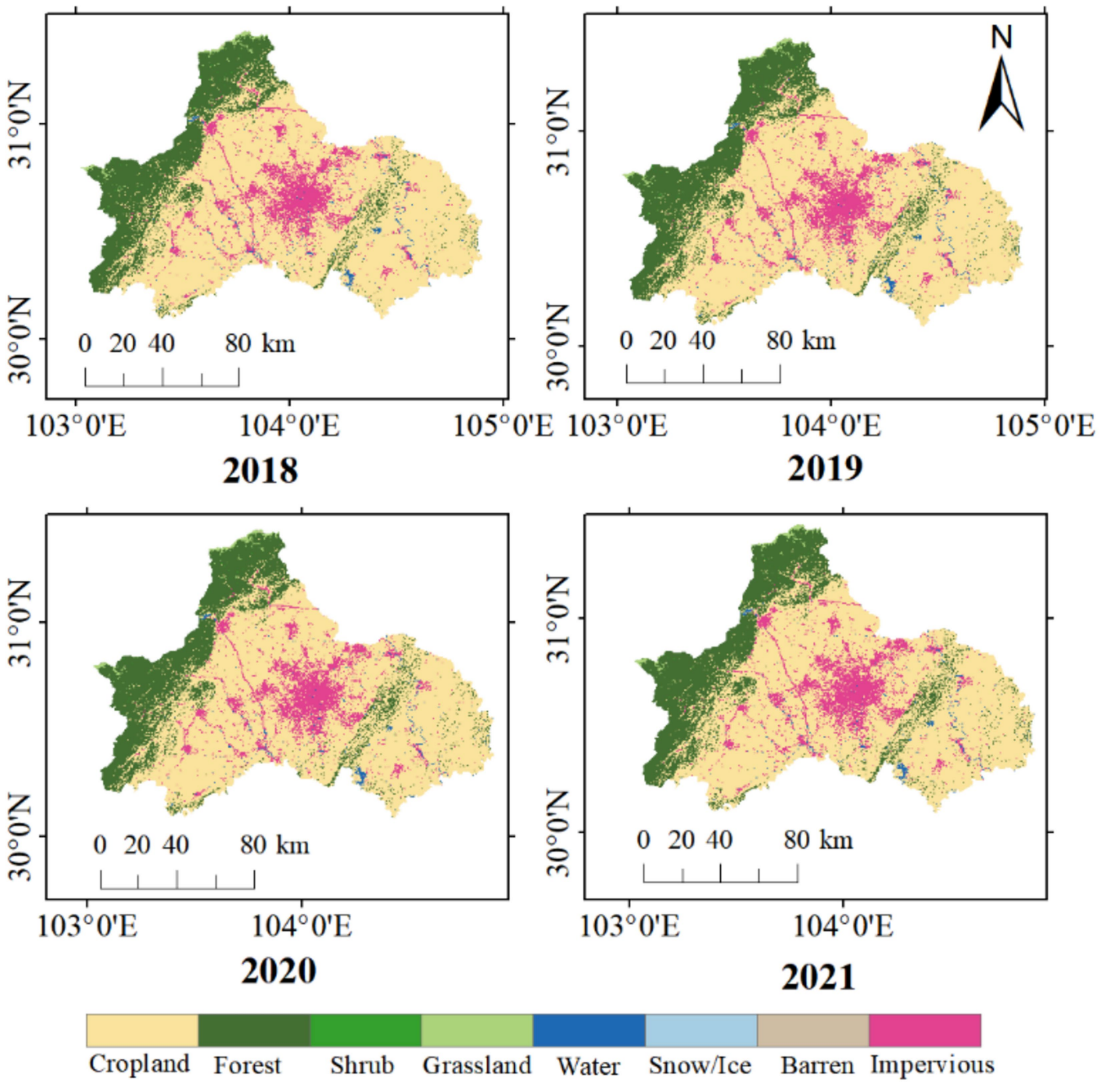
Surface cover type in Chengdu from 2018 to 2021.

Based on our analysis, there was noticeable spatial variation in land cover type in the study region (Figure 3). In general, the lowest levels of green vegetation cover were observed in city center (surface cover type: impervious). The southwest corridor of the city was covered in forest, while most of the city was covered by crop land. There was no observable temporal change in land cover type during the study period (*p* value for trend: 0.28).

**Figure 4:**
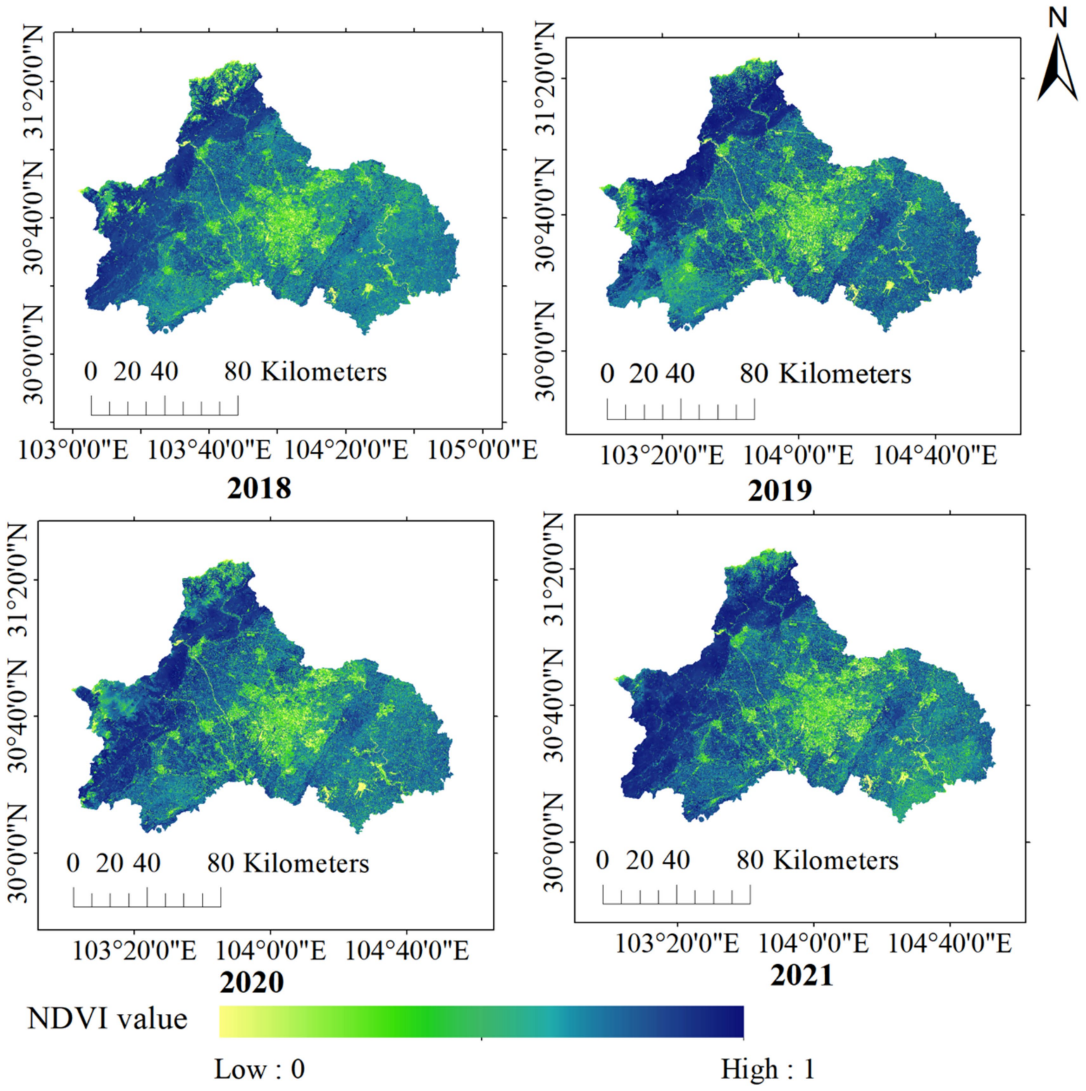
Annual average greenness coverage in Chengdu from 2018 to 2021.

Annual NDVI was used to analyze the greenness coverage in Chengdu plain. NDVI varied spatially across Chengdu (Figure 4), specifically, NDVI was higher in the northwest corridor and lower in the metropolitan region in the central plain. Between 2018 and 2021, NDVI exhibited no statistically significant temporal change in the metropolitan core area (Figure 4).

At the district level, districts in the west and the northwest showed higher greenness coverage (Supplementary Figure S1). In particular, Pengzhou, Dujiangyan, Chongzhou, Dayi, Qionglai all exhibited relatively high NDVI values. Qionglai District had the highest NDVI value (0.7715) and greenness coverage (86.15%) in 2018, and Wuhou had the lowest NDVI value (0.4185) and greenness coverage (33.38%). In 2019, Dujiangyan had the highest NDVI value (0.7738) and greenness coverage rate (86.50%), while Wuhou still had the lowest NDVI value (0.4222), with a greenness coverage rate of 33.94%.

### Odds ratio between NDVI and ASD prevalence

Logistic regression on NDVI against ASD screening result yielded no significant association (Table 2). While no statistical significance was found, the odds ratios do confirm earlier understanding that males were more likely to be diagnosed with ASD than females (55% ~ 60% increased odds). Meanwhile, older children had higher odds to receive positive ASD diagnoses than younger children (3% increased odds per month).

**Table 2:** Summary of odds ratio (95% CI).

Year	Gender^#^	Age (month)	NDVI
Four-year average (2018–2021)	1.60 (0.70–3.68)	1.03 (0.93–1.14)	2.94 (0.17–49.84)
2021	1.59 (0.69–3.64)	1.03 (0.93–1.14)	1.63 (0.12–22.28)
2020	1.55 (0.68–3.57)	1.03 (0.93–1.14)	3.85 (0.30–50.23)
2019	1.58 (0.69–3.62)	1.03 (0.93–1.14)	2.20 (0.20–23.94)
2018	1.58 (0.69–3.62)	1.03 (0.93–1.14)	1.38 (0.10–18.74)

Primary outcome of interest is whether or not the ASD diagnosis is positive and the primary independent variable of interest is annual mean NDVI. Model adjusted for age, gender, average PM_2.5_, PM_10_, O_3_, SO_2_, NO_2_, CO, temperature and relative humidity. #Reference for gender is “female.” Significance level: *p value <0.05; **p value <0.005; ***p value <0.001.

Additional stratified analysis based on gender yielded non-significant association for both males and females, however, the estimates suggested higher odds for females (Table 3), a result similar to previously reported in U. S. children (30). Additional analysis of buffered NDVI (radii of 50 m, 100 m, 500 m, and 1,000 m) did not show significant association (Supplementary Table S3).

**Table 3:** Summary of regression results for ASD diagnosis and annual mean NDVI, stratified by gender.

Male		Female	
Four-year average 2018–2021	1.72 (0.05–65.20)	Four-year average 2018–2021	6.07 (0.07–559.82)
2021	1.00 (0.98–1.02)	2021	8.02 (0.11–611.36)
2020	1.00 (0.99–1.02)	2020	4.92 (0.09–265.80)
2019	2.03 (0.10–40.50)	2019	3.68 (0.07–194.96)
2018	1.23 (0.05–32.08)	2018	1.85 (0.02–149.80)

Primary outcome of interest is whether or not the ASD diagnosis is positive and the primary independent variable of interest is annual mean NDVI. Model adjusted for age, gender, average PM_2.5_, PM_10_, O_3_, SO_2_, NO_2_, CO, temperature and relative humidity. #Reference for gender is “female.” Significance level: *p value <0.05; **p value <0.005; ***p value <0.001.

## Discussion

In this study, we recruited 13,458 children from local maternal and child healthcare network during routine child’s healthcare check to assess the potential relationship between ASD outcome and greenness exposure, as represented by NDVI. The result indicated that the ASD prevalence among the study population was 0.55%. This study did not find statistically significant association between residential outdoor greenness exposure and early childhood ASD diagnoses after adjusting for air pollution exposure and weather influence. This lack of association could be due to (1) study population being too young for definitive ASD diagnosis (low ASD prevalence), and (2) exposure window being too short to establish an association between residential greenness and ASD outcome. Nonetheless, data from city-wide screening based on a multi-center system provides real-world evidence for understanding the relationship between greenness exposure and ASD diagnosis.

### Diagnosis age and childhood ASD

ASD, as a biological neurodevelopmental disorder, may occur alone, but more often co-occurs with other NDD (neurodevelopmental disorders), medical, psychiatric, or genetic disorders (31). Because of the type and severity of ASD symptoms vary widely and can change with age, diagnosing ASD is a fairly complex and arduous process (32). In younger children, especially those under the age of 3, diagnosing ASD can be challenging due to subtle symptoms that might not be fully apparent or distinguishable from typical developmental variations (33, 49). Studies have shown that babies younger than 16 months or before the age of 3 years are at a higher risk of misdiagnosis (34, 35). Children in our study were aged 0 to 52 months, and more than 99 per cent of the total number of children aged 36 months or less. Because most (94%) of our participants were younger than 2 years old, this may explain the lack of significant association between greenness and ASD as it is more difficult to diagnose ASD for children under 2 years of age. Child ASD development is a lengthy process with unclear developmental mechanism. Younger children could have less obvious symptoms and thus less positive diagnosis. Therefore, subsequent research on the relationship between greenery exposure and childhood autism needs to be further adapted and improved.

### Observation time and childhood ASD

Our study was an explorative attempt to use a two-stage screening process designed to capture ASD symptoms early in childhood. Because the types of autism symptoms vary widely and change with age, long-term studies often span several years to capture developmental progress, while short-term studies typically evaluate immediate responses to interventions, such as behavioral therapy or educational support. Short-term studies, like our current study, typically focus on the early developmental trajectories of children with autism, particularly in the context of early intervention. They track changes in behavioral, communication and cognitive skills during critical early childhood periods (36). These studies are critical for guiding early intervention strategies that have a significant positive impact on children’s cognitive and social development (37). However, the link between greenness exposure and ASD outcomes could take longer to form, thus requiring a longer study period.

Early childhood is a critical developmental stage in a child’s life, the relationship between childhood development and greenness exposure has been a growing area of interest in recent years. Multiple studies in the past have shown that exposure to urban green space has benefits for mental and physical health of children and adolescents (38–40), especially with positive effect on attention, memory, and executive function (41) as well as faster cognitive development (42). At the societal level, higher greenness exposure has been linked to improved emotional regulation and behavioral outcomes in children and lower likelihood of developing psychiatric disorders such as schizophrenia (39, 43, 44). The study area Chengdu is a rapidly urbanizing megacity. Exposure to urban green space is local residents’ primary contact with nature. Therefore, using real-world evidence to assess the environmental factors for ASD development and diagnosis could provide scientific evidence for resource distribution and early intervention.

### Strengths and limitations

Our analysis benefited from a relatively large population recruited from a city-wide maternal and childcare network, which allowed us to survey a sizable and relatively representative study population. Our analysis was also strengthened by person-specific exposure to greenness and regression analysis controlled for potential confounding from air pollution and weather.

However, our analysis was limited by a few factors. Although the ASD screening process included a large population, participation in the environmental questionnaire was voluntary and thus could have resulted in self-selective subpopulation (based on willingness to fill out the environmental questionnaire). Thus, our final study population of 4,362 may not reflect the general population, which limits the scope of inference for our study findings. Additionally, a time lag between potential exposure effect and ASD diagnosis could be longer than our study period, therefore, the analytical result could be limited by available data and the resulting association is non-significant. Furthermore, our analysis only considered outdoor greenness, yet indoor greenness exposure could be a confounder that affects the association in reality. Similarly, our data did not include information on socioeconomic status, which could be potential confounder that attenuates the regression results. We only adjusted for potential confounding from air pollution and weather influence, however, we did not explore any possible interaction between these two confounders and greenness exposure; analyzing such interaction could be informative to the combined effect of these exposures. Lastly, the accuracy of remote sensing data is affected by a number of factors such as obstructions between satellite and ground objects (such as cloud coverage) and time differences in satellite images; to obtain the data of the entire Chengdu area, the images of different areas taken at different times are spliced together. Due to the slight difference in shooting time for the different areas on the composing image, there may be uncontrollable differences in vegetation coverage, but it does not affect the overall vegetation coverage calculation and analysis on longer time scales.

## Conclusion

In this primary study, we investigated the relationship between greenness exposure and childhood ASD using real-world data. By screening 13,458 children aged 0–52 months and collecting both clinical and environmental data in Chengdu, China, we estimated ASD prevalence to be 0.55% but did not find statistically significant association between greenness exposure and childhood ASD. Results from this study provide foundational understanding of the regional ASD prevalence among young children and scientific evidence for further research in examining the mechanism underlying ASD development.

## Data Availability

The raw data supporting the conclusions of this article will be made available by the authors upon request.

## References

[1] Li D Larsen L Yang Y Wang L Zhai Y Sullivan WC. Exposure to nature for children with autism spectrum disorder: benefits, caveats, and barriers. Health Place. (2019) 55:71–9. doi: 10.1016/j.healthplace.2018.11.005, PMID: 30503683

[2] The Newcastle upon Tyne Hospitals. (2025). Understanding and supporting the social interaction of autistic children and young people. Available online at: https://www.newcastle-hospitals.nhs.uk/resources/understanding-and-supporting-the-social-interaction-of-autistic-children-and-young-people/

[3] Zeidan J Fombonne E Scorah J Ibrahim A Durkin MS Saxena S. Global prevalence of autism: a systematic review update. Autism Res. (2022) 15:778–90. doi: 10.1002/aur.2696, PMID: 35238171 PMC9310578

[4] Chiarotti F Venerosi A. Epidemiology of autism Spectrum disorders: a review of worldwide prevalence estimates since 2014. Brain Sci. (2020) 10:27. doi: 10.3390/brainsci10050274, PMID: 32370097 PMC7288022

[5] Cuesta-Gomez JL De la Fuente-Anuncibay RR Vidriales-Fernandez R Ortega-Camarero MT. The quality of life of people with ASD through physical activity and sports. Heliyon. (2022) 8:e09193. doi: 10.1016/j.heliyon.2022.e09193 35368544 PMC8966138

[6] Wei H Li Y Zhang Y Luo J Wang S Dong Q. Awareness and knowledge of autism spectrum disorder in Western China: promoting early identification and intervention. Front Psych. (2022) 13:970611. doi: 10.3389/fpsyt.2022.970611, PMID: 36440386 PMC9686393

[7] Sun X Allison C Matthews FE Sharp SJ Auyeung B Baron-Cohen S. Prevalence of autism in mainland China, Hong Kong and Taiwan: a systematic review and meta-analysis. Mol Autism. (2013) 4:7. doi: 10.1186/2040-2392-4-7, PMID: 23570419 PMC3643868

[8] Zhou H Xu X Yan W Zou X Wu L Luo X. Prevalence of autism Spectrum disorder in China: a Nationwide multi-center population-based study among children aged 6 to 12 years. Neurosci Bull. (2020) 36:961–71. doi: 10.1007/s12264-020-00530-6, PMID: 32607739 PMC7475160

[9] Larson LR Barger B Ogletree S Torquati J Rosenberg S Gaither CJ. Gray space and green space proximity associated with higher anxiety in youth with autism. Health Place. (2018) 53:94–102. doi: 10.1016/j.healthplace.2018.07.006, PMID: 30059898

[10] Wu J Jackson L. Inverse relationship between urban green space and childhood autism in California elementary school districts. Environ Int. (2017) 107:140–6. doi: 10.1016/j.envint.2017.07.010, PMID: 28735150 PMC6104398

[11] Kondo MC Fluehr JM McKeon T Branas CC. Urban green space and its impact on human health. Int J Environ Res Public Health. (2018) 15. doi: 10.3390/ijerph15030445, PMID: 29510520 PMC5876990

[12] Sarkar C Webster C Gallacher J. Residential greenness and prevalence of major depressive disorders: a cross-sectional, observational, associational study of 94 879 adult UK biobank participants. Lancet Planet Health. (2018) 2:e162–73. doi: 10.1016/S2542-5196(18)30051-2, PMID: 29615217

[13] Chengdu Bureau of Statistics (2018). Chengdu statistical yearbook. China Statistics Press. Available online at: http://cdstats.chengdu.gov.cn/tjgzxxw/xhtml/tjxx_content.html?id=123764&channel= (Accessed March 4, 2022).

[14] Chengdu Bureau of Statistics (2019). Chengdu statistical yearbook. China Statistics Press. Available online at: http://cdstats.chengdu.gov.cn/tjgzxxw/xhtml/tjxx_content.html?id=179930&channel= (Accessed March 4, 2022).

[15] Jiang L Liu Y Wu S Yang C. Analyzing ecological environment change and associated driving factors in China based on NDVI time series data. Ecol Indic. (2021) 129:7933. doi: 10.1016/j.ecolind.2021.107933

[16] Reid CE Kubzansky LD Li J Shmool JL Clougherty JE. It's not easy assessing greenness: a comparison of NDVI datasets and neighborhood types and their associations with self-rated health in new York City. Health Place. (2018) 54:92–101. doi: 10.1016/j.healthplace.2018.09.005, PMID: 30248597

[17] Ding YL Zhao K Zheng XM Jiang T. Temporal dynamics of spatial heterogeneity over cropland quantified by time-series NDVI, near infrared and red reflectance of Landsat 8 OLI imagery. Int J Appl Earth Obs Geoinf. (2014) 30:139–45. doi: 10.1016/j.jag.2014.01.009

[18] Ke Y Im J Lee J Gong H Ryu Y. Characteristics of Landsat 8 OLI-derived NDVI by comparison with multiple satellite sensors and in-situ observations. Remote Sens Environ. (2015) 164:298–313. doi: 10.1016/j.rse.2015.04.004

[19] Yang J. Huang X. (2024). The 30 m annual land cover datasets and its dynamics in China from 1985 to 2023. Available online at: https://zenodo.org/records/12779975.

[20] ArcGIS. (2021). ArcGIS. Available online at: https://www.arcgis.com

[21] Carter SA Rahman MM Lin JC Shu YH Chow T Yu X. In utero exposure to near-roadway air pollution and autism spectrum disorder in children. Environ Int. (2022) 158:106898. doi: 10.1016/j.envint.2021.106898, PMID: 34627014 PMC8688235

[22] Ibrahim MF Hod R Nawi AM Sahani M. Association between ambient air pollution and childhood respiratory diseases in low- and middle-income Asian countries: a systematic review. Atmos Environ. (2021) 256:118422. doi: 10.1016/j.atmosenv.2021.118422

[23] Kerin T Volk H Li W Lurmann F Eckel S McConnell R. Association between air pollution exposure, cognitive and adaptive function, and ASD severity among children with autism Spectrum disorder. J Autism Dev Disord. (2018) 48:137–50. doi: 10.1007/s10803-017-3304-0, PMID: 28921105 PMC5764162

[24] China National Environmental Monitoring Centre. (2022). Air quality report. Available online at: http://www.cnemc.cn/ (Accessed September 1, 2022).

[25] National Oceanic and Atmospheric Administration. (2022). NOMADS Data at NCEP. Available online at: https://nomads.ncep.noaa.gov/ (Accessed September 1, 2022)

[26] Zhan Y Luo Y Deng X. Spatiotemporal prediction of continuous daily PM2.5 concentrations across China using a spatially explicit machine learning algorithm. Atmos Environ. (2017) 155:129–39. doi: 10.1016/j.atmosenv.2017.02.023

[27] Zhan Y Luo Y Deng X Grieneisen ML Zhang M Di B. Spatiotemporal prediction of daily ambient ozone levels across China using random forest for human exposure assessment. Environ Pollut. (2018a) 233:464–73. doi: 10.1016/j.envpol.2017.10.029, PMID: 29101889

[28] Zhan Y Luo Y Deng X Zhang K Zhang M Grieneisen ML. Satellite-based estimates of daily NO(2) exposure in China using hybrid random forest and spatiotemporal kriging model. Environ Sci Technol. (2018b) 52:4180–9. doi: 10.1021/acs.est.7b05669 29544242

[29] IBM Corp. IBM SPSS statistics for windows, version 24.0 IBM Corp (2016).

[30] Fong KC Hart JE James P. A review of epidemiologic studies on greenness and health: updated literature through 2017. Curr Environ Health Rep. (2018) 5:77–87. doi: 10.1007/s40572-018-0179-y, PMID: 29392643 PMC5878143

[31] Genovese A Butler MG. The autism spectrum: behavioral, psychiatric and genetic associations. Genes. (2023) 14:677. doi: 10.3390/genes14030677 36980949 PMC10048473

[32] Lord C Elsabbagh M Baird G Veenstra-Vanderweele J. Autism spectrum disorder. Lancet. (2018) 392:508–20. doi: 10.1016/s0140-6736(18)31129-2, PMID: 30078460 PMC7398158

[33] Shattuck PT Durkin M Maenner M Newschaffer C Mandell DS Wiggins L. (2009). Timing of identification among children with an autism spectrum disorder: findings from a population-based surveillance study. Journal of the American Academy of Child and Adolescent Psychiatry, 48, 474–483. doi: 10.1097/CHI.0b013e31819b3848 19318992 PMC3188985

[34] Kleinman JM Robins DL Ventola PE Pandey J Boorstein HC Esser EL. The modified checklist for autism in toddlers: a follow-up study investigating the early detection of autism spectrum disorders. J Autism Dev Disord. (2008) 38:827–39. doi: 10.1007/s10803-007-0450-9, PMID: 17882539 PMC3612529

[35] Sumner E Leonard HC Hill EL. Comparing attention to socially-relevant stimuli in autism Spectrum disorder and developmental coordination disorder. J Abnorm Child Psychol. (2018) 46:1717–29. doi: 10.1007/s10802-017-0393-3, PMID: 29313185 PMC6208873

[36] Hadzic S Biscevic I Memisevic H. Advancements in early intervention for children with autism: a five-year review. Multidisciplinarni Pristupi u Edukaciji i Rehabilitaciji. (2024) 6:44–56. doi: 10.59519/mper6105

[37] Daniolou S Pandis N Znoj H. The efficacy of early interventions for children with autism Spectrum disorders: a systematic review and Meta-analysis. J Clin Med. (2022) 11:5100. doi: 10.3390/jcm11175100, PMID: 36079029 PMC9457367

[38] Barboza EP Cirach M Khomenko S Iungman T Mueller N Barrera-Gómez J. Green space and mortality in European cities: a health impact assessment study. Lancet Planet Health. (2021) 5:e718–30. doi: 10.1016/S2542-5196(21)00229-1, PMID: 34627476

[39] Liu Y Wang R Xiao Y Huang B Chen H Li Z. Exploring the linkage between greenness exposure and depression among Chinese people: mediating roles of physical activity, stress and social cohesion and moderating role of urbanicity. Health Place. (2019) 58:102168. doi: 10.1016/j.healthplace.2019.102168, PMID: 31325813

[40] Pérez-Crespo L Prats-Uribe A Tobias A Duran-Tauleria E Coronado R Hervás A. Temporal and geographical variability of prevalence and incidence of autism Spectrum disorder diagnoses in children in Catalonia, Spain. Autism Res. (2019) 12:1693–705. doi: 10.1002/aur.2172, PMID: 31317678 PMC6900126

[41] Jimenez MP DeVille NV Elliott EG Schiff JE Wilt GE Hart JE. Associations between nature exposure and health: a review of the evidence. Int J Environ Res Public Health. (2021) 18:4790. doi: 10.3390/ijerph18094790, PMID: 33946197 PMC8125471

[42] Dadvand P Nieuwenhuijsen MJ Esnaola M Forns J Basagaña X Alvarez-Pedrerol M. Green spaces and cognitive development in primary schoolchildren. Proc Natl Acad Sci USA. (2015) 112:7937–42. doi: 10.1073/pnas.1503402112, PMID: 26080420 PMC4491800

[43] Engemann K Pedersen CB Arge L Tsirogiannis C Mortensen PB Svenning JC. Residential green space in childhood is associated with lower risk of psychiatric disorders from adolescence into adulthood. Proc Natl Acad Sci USA. (2019) 116:5188–93. doi: 10.1073/pnas.1807504116, PMID: 30804178 PMC6421415

[44] Mygind L Kjeldsted E Hartmeyer R Mygind E Bølling M Bentsen P. Mental, physical and social health benefits of immersive nature-experience for children and adolescents: a systematic review and quality assessment of the evidence. Health Place. (2019) 58:18. doi: 10.1016/j.healthplace.2019.05.014 31220797

[45] Cheroni C Caporale N Testa G. Autism spectrum disorder at the crossroad between genes and environment: contributions, convergences, and interactions in ASD developmental pathophysiology. Mol Autism. (2020) 11:69. doi: 10.1186/s13229-1 32912338 PMC7488083

[46] Creem AN Rodriguez KA Hillhouse BJ Lee R Leaf JB. Early intensive behavioral intervention for autism Spectrum disorder In: Matson JL, editor. Handbook of clinical child psychology: Integrating theory and research into practice. Cham: Springer International Publishing (2023). 635–57.

[47] Dominski FH Branco JHL Buonanno G Stabile L Silva MGd Andrade A. Effects of air pollution on health: a mapping review of systematic reviews and meta-analyses. Environ Res. (2021) 201:111487. doi: 10.1016/j.envres.2021.111487 34116013

[48] Frye RE Cakir J Rose S Delhey L Bennuri SC Tippett M. Prenatal air pollution influences neurodevelopment and behavior in autism spectrum disorder by modulating mitochondrial physiology. Mol Psychiatry. (2021) 26:1561–77. doi: 10.1038/s41380-020-00885-2, PMID: 32963337 PMC8159748

[49] Harstad E Hanson E Brewster SJ DePillis R Milliken AL Aberbach G. Persistence of autism Spectrum disorder from early childhood through school age. JAMA Pediatr. (2023) 177:1197–205. doi: 10.1001/jamapediatrics.2023.4003, PMID: 37782510 PMC10546296

[50] Lai MC Kassee C Besney R Bonato S Hull L Mandy W. Prevalence of co-occurring mental health diagnoses in the autism population: a systematic review and meta-analysis. Lancet Psychiatry. (2019) 6:819–29. doi: 10.1016/s2215-0366(19)30289-5, PMID: 31447415

[51] R-Forge. R: A language and environment for statistical computing. Vienna, Austria: R Foundation for Statistical Computing (2018).

[52] van ‘t Hof M Tisseur C van Berckelear-Onnes I van Nieuwenhuyzen A Daniels AM Deen M. Age at autism spectrum disorder diagnosis: a systematic review and meta-analysis from 2012 to 2019. Autism. (2021) 25:862–73. doi: 10.1177/1362361320971107 33213190

[53] Yoo EH Roberts JE Eum Y Li X Konty K. Exposure to urban green space may both promote and harm mental health in socially vulnerable neighborhoods: a neighborhood-scale analysis in new York City. Environ Res. (2022) 204:112292. doi: 10.1016/j.envres.2021.112292, PMID: 34728238

[54] Lavelle TA Weinstein MC Newhouse JP Munir K Kuhlthau KA Prosser LA. (2014). Economic burden of childhood autism spectrum disorders. Pediatrics, 133, e520–e529. doi: 10.1542/peds.2013-0763 24515505 PMC7034397

