# Supplementary material for: Real-world evidence from a multi-center pediatric network: greenness exposure and autism spectrum disorder in urban China
Source: Front Public Health. 2025 Sep 17;13:1666873. doi: 10.3389/fpubh.2025.1666873 (PMC12484007; doi:10.3389/fpubh.2025.1666873)
Supplement: Supplementary file 1 [file Data_Sheet_1.pdf]

Supplemental Materials

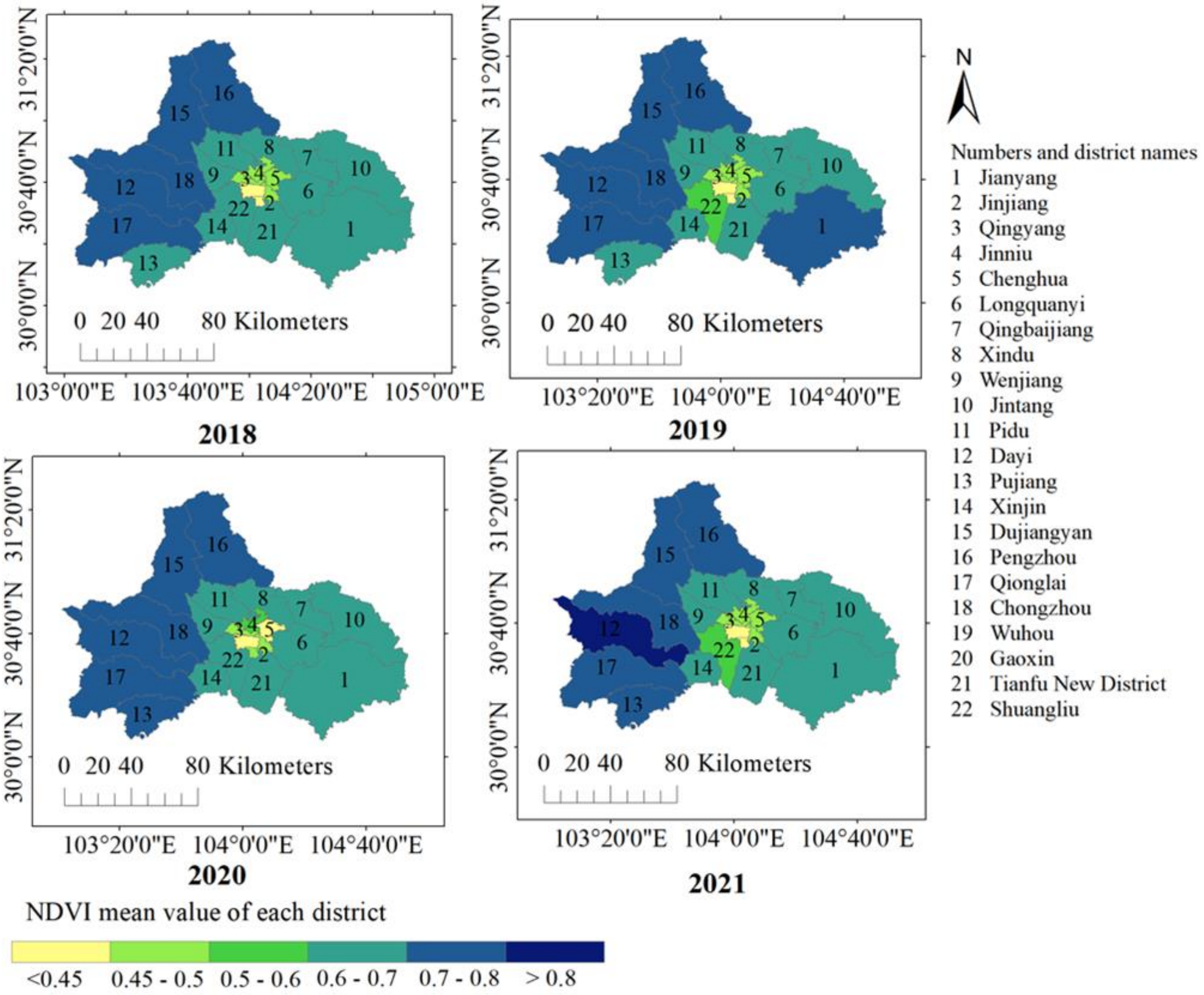

Figure S1. Annual mean NDVI for each district in Chengdu from 2018 to 2021.

Table S1. List of participating women and childcare hospitals

Participating Hospital	Corresponding District/County/City*
Chenghua Women and Children Healthcare Center	Chenghua District
Chongzhou Women and Children Healthcare Center	Chongzhou City
Dayi Women and Children Healthcare Center	Dayi County
Dujiangyan Women and Children Healthcare Center	Dujiangyan City
Jianyang Women and Children Healthcare Center	Jianyang City
Jinjiang Women and Children Healthcare Center	Jinjiang District
Jinniu Women and Children Healthcare Center	Jinniu District
Jintang Women and Children Healthcare Center	Jintang County
Longquanyi Women and Children Healthcare Center	Longquanyi District
Pengzhou Women and Children Healthcare Center	Pengzhou City
Pidu Women and Children Healthcare Center	Pidu District
Pujiang Women and Children Healthcare Center	Pujiang County
Qingbaijiang Women and Children Healthcare Center	Qingbaijiang District
Qionglai Women and Children Healthcare Center	Qionglai City
Shuangliu Women and Children Healthcare Center	Shuangliu District
Wenjiang Women and Children Healthcare Center	Wenjiang District
Wuhou Women and Children Healthcare Center	Wuhou District
Xindu Women and Children Healthcare Center	Xindu District
Xinjin Women and Children Healthcare Center	Xinjin District
Huayang Community HealthCare Center	Tianfu New Area**

*City here refers to county-level administrative district that is part of metropolitan Chengdu. **Tianfu New Area is a special administrative district.

Table S2. Summary Information of Four Screening Tools

Screen Tool Name	Age group	Details
Autism warning sign (AWS)	0-6 years old	adaptation of Chinese warning sign checklist (WSC), a 44-indicator list established by a Chinese expert group for monitoring psychological and behavioral development; includes indicators for language development and social interaction from WSC because doctors are most likely to notice language deficiency, social behavior and communication issues
Social behaviour observational assessment (SB)	0-1 years old	an adaptation of WSC for 12-months-old. SB contains two indicators: imitation of conventional gestures (waving goodbye or claps) and name response. ASD patients often

		exhibit non-verbal behaviour at young age and persist over time, with difficulty in imitating common social gestures (such as goodbye or clap), response to their names, pointing with a finger, interest in others and joint attention
Chinese-validated version of the Checklist for Autism in Toddlers(CHAT23)	18-24 months old	Part A of CHAT23 is composed of 23 questions for caregivers, and positive result for 6 out of the 23 indicators or 2 out of the 7 core indicators results in a positive screening result. Part B is a short observation that consists of 4 questions evaluated by doctors during face-to-face interviews with children. Two or more abnormal results result in a positive screening result. Failure in either part A or part B results in a positive screening result.
The Autism Behaviour Checklist (ABC)	18 months -35 years old	The Chinese version of ABC is a 5-part and 57 indicators questionnaire covering sensory behaviour, social relating, body and object use, language and communication skills, and social and adaptive skills. A total score above 53 implies a positive screening result.

Table S3. Summary of regression results from supplementary model with annual mean NDVI at radii of 50m, 100m, 500m and 1000m from the reported residential address.

	Year	Buffer Radii (m)	Gender (Male)	Age (month)	NDVI
Supplementary models	Four-Year Average 2018-2021	50 m	1.62(0.71-3.74)	0.99(0.89-1.12)	0.54(0.12-2.39)
		100 m	1.63(0.71-3.74)	1.00(0.89-1.12)	0.43(0.10-1.95)
		500 m	1.62(0.71-3.72)	0.99(0.88-1.12)	0.78(0.18-3.40)
		1000 m	1.62(0.71-3.72)	0.99(0.88-1.12)	0.53(0.11-2.65)
	2021	50 m	1.33(0.42-4.22)	1.08(0.93-1.27)	2.45(0.08-71.54)
		100 m	1.32(0.42-4.18)	1.08(0.93-1.26)	0.45(0.01-20.60)
		500 m	1.66(0.48-5.70)	1.11(0.94-1.30)	9.42(0.15-598.29)
		1000 m	2.28(0.44-11.84)	1.12(0.93-1.34)	6.28(0.03-1480.00)
	2020	50 m	1.30(0.41-4.12)	1.08(0.93-1.25)	5.17(0.19-144.59)
		100 m	1.30(0.41-4.11)	1.08(0.93-1.25)	2.06(0.06-74.47)
		500 m	1.64(0.48-5.64)	1.10(0.96-1.27)	9.21(0.14-590.69)
		1000 m	2.14(0.41-11.21)	1.10(0.93-1.29)	9.55(0.04-2353.47)
	2019	50 m	1.28(0.41-4.07)	1.08(0.92-1.26)	2.09(0.08-57.31)
		100 m	1.28(0.40-4.06)	1.08(0.92-1.26)	1.56(0.04-59.82)
		500 m	1.66(0.48-5.70)	1.11(0.95-1.29)	8.81(0.12-645.86)
		1000m	1.66(0.48-5.70)	1.11(0.95-1.29)	8.81(0.12-645.86)
	2018	50 m	1.28(0.40-4.04)	1.08(0.93-1.26)	3.47(0.11-106.64)
		100m	1.28(0.41-4.07)	1.08(0.93-1.26)	0.74(0.02-32.33)
		500 m	1.67(0.48-5.73)	1.11(0.94-1.29)	6.06(0.08-447.59)
		1000 m	2.29(0.44-11.95)	1.11(0.92-1.34)	10.22(0.03-3539.59)

Formula

FVC is obtained based on the following formula:

$$\mathrm{FVC}=\frac{\mathrm{NDVI}-\mathrm{NDVI}_{\mathrm{soil}}}{\mathrm{NDVI}_{\mathrm{veg}}-\mathrm{NDVI}_{\mathrm{soil}}} \tag{S1}$$

where NDVI is the NDVI value of the target pixel; NDVIveg is the NDVI value of the full vegetation pixel; NDVIsoil is the NDVI value of the bare soil pixel.

NDVIveg and NDVIsoil are obtained based on the following formula:

$$\mathrm{FVC}=\frac{\mathrm{NDVI}-\mathrm{NDVI}_{\mathrm{soil}}}{\mathrm{NDVI}_{\mathrm{veg}}-\mathrm{NDVI}_{\mathrm{soil}}} \tag{S2}$$

$$\mathrm{NDVI}_{\mathrm{soil}}=\frac{(1-\mathrm{FVC}_{\mathrm{min}})\times\mathrm{NDVI}_{\mathrm{max}}-(1-\mathrm{FVC}_{\mathrm{max}})\times\mathrm{NDVI}_{\mathrm{min}}}{\mathrm{FVC}_{\mathrm{max}}-\mathrm{FVC}_{\mathrm{min}}} \tag{S3}$$

can be approximately taken in the region, then FVC can be approximately calculated by formula (6):

$$\mathrm{FVC}=\frac{\mathrm{NDVI}-\mathrm{NDVI}_{\mathrm{min}}}{\mathrm{NDVI}_{\mathrm{min}}-\mathrm{NDVI}_{\mathrm{max}}} \tag{S4}$$
